# Early Screening for Developmental Dysplasia of the Hip: Sonographic Reference Values, Risk Factors, and Treatment Considerations

**DOI:** 10.3390/ijns11030081

**Published:** 2025-09-19

**Authors:** Bjoern Vogt, Stella S. Tureck, Georg Gosheger, Adrien Frommer, Andrea Laufer, Henning Tretow, Robert Roedl, Gregor Toporowski

**Affiliations:** 1 Pediatric Orthopedics, Deformity Reconstruction and Foot Surgery, Muenster University Hospital, Albert-Schweitzer-Campus 1, 48149 Muenster, Germany; bjoern.vogt@ukmuenster.de (B.V.); stellasophia.tureck@ukmuenster.de (S.S.T.); adrien.frommer@ukmuenster.de (A.F.); andrea.laufer@ukmuenster.de (A.L.); henning.tretow@ukmuenster.de (H.T.); robert.roedl@ukmuenster.de (R.R.); 2 General Orthopedics and Tumor Orthopedics, Muenster University Hospital, Albert-Schweitzer-Campus 1, 48149 Muenster, Germany; georg.gosheger@ukmuenster.de

**Keywords:** developmental dysplasia of the hip, DDH, hip dislocation, Graf method, mass screening, ultrasonography, infant, newborn, risk factors

## Abstract

Developmental dysplasia of the hip (DDH) is a common neonatal musculoskeletal disorder. In Germany, sonographic screening is recommended at 1–10 days of life for neonates with specific risk factors. This study aims to determine reference values for early sonographic screening and to evaluate associated risk factors. Between 2007 and 2022, 3383 neonates (6766 hips) underwent hip ultrasound according to Graf. Of these, 967 neonates were screened universally (2007–2015) and 1900 based on predefined risk factors (2015–2022). DDH was defined as ≥type IIc, according to Graf. A subgroup of 20 neonates with borderline alpha angles (51–52°) was followed up after 3–6 weeks. The mean alpha angle was 61.2° ± 5.3° (range 50.5–71.9°), and beta angle 70.8° ± 8.6° (range 53.6–88.0°). DDH prevalence was 2.5% in the universal and 3.2% in the risk-based cohort (*p* = 0.350). Logistic regression revealed associations with abnormal birth presentation (OR = 3.09, *p* < 0.001) and female sex (OR = 3.77, *p* < 0.001), not with Cesarean section or familial predisposition. In the follow-up subgroup, all hips showed a sufficient maturation to an alpha angle of 61.0° (range 57–66°). This study provides reference values for early DDH screening and confirms abnormal birth presentation and female sex as relevant risk factors.

## 1. Introduction

Developmental dysplasia of the hip (DDH) is a common musculoskeletal condition in neonates, with a prevalence of 0.5–6% in Europe [1,2,3]. It is characterized by abnormal development of the hip joint, in which the femoral head is not sufficiently covered by the acetabulum. This may result in varying degrees of mechanical instability, including acetabular dysplasia, subluxation, or complete dislocation of the femoral head [1,2,3]. The most significant postnatal maturation of the hip joint is described within the first three months after birth and can be actively promoted through conservative interventions, such as the Pavlik harness or similar orthoses [4,5]. This non-invasive treatment has been shown to effectively support hip joint development and prevent secondary osteoarthritis due to hip dysplasia [5,6].

Since 1996, Germany has implemented a nationwide universal sonography screening program for DDH at 4–6 weeks of age, which is now an established component of the national clinical guideline [7,8,9]. Since its introduction, the prevalence of DDH requiring surgical treatment—both acetabuloplasty to improve femoral head coverage and open reduction of the hip—has significantly declined [9,10,11,12]. Nevertheless, the effectiveness of generalized sonographic DDH screening is questioned in many countries. A key concern is that normal findings detected shortly after birth do not provide DDH at a later stage [2]. Additionally, clinical screening is often considered sufficient, as sonography is thought to increase the risk of overtreatment [2,13]. However, clinical examination alone is only reliable in detecting severe cases of DDH, as it primarily identifies unstable or dislocated hips rather than subtle dysplastic changes [14].

As the potential for postnatal hip maturation is greater in younger patients, the German guideline recommends performing sonographic DDH screening in the early stage within the first days after birth if specific risk factors, such as a positive family history and breech presentation at birth, are present [7,15,16]. Foot deformities are also identified as a risk factor for DDH in the guideline, although the literature presents conflicting evidence regarding their relevance [17,18,19]. Additionally, other factors associated with DDH, such as oligohydramnios or twin pregnancy, have been reported but are not explicitly included in the guideline [20,21]. While twin pregnancy is not considered an independent risk factor, associated intrauterine mechanical conditions, such as breech presentation or limited mobility, may contribute to DDH [21,22,23].

In Austria and other European countries, general sonographic DDH screening is already performed additionally within the first week after birth [24]. The most commonly used sonographic DDH screening method in Europe is the Graf technique [25,26]. Reference values for the Graf technique have been established for infants aged 4–6 weeks [7]; however, these do not apply to early sonographic DDH screening due to the ongoing hip maturation between the first days and 4-6 weeks after birth. Therefore, it is essential to establish reference values for early screening to facilitate accurate assessment and informed treatment decisions.

The objectives of this study were (1) to determine reference values for early sonographic DDH screening and (2) to assess which risk factors are associated with DDH in early sonographic DDH screening.

## 2. Materials and Methods

### 2.1. Study Design and Population

This study retrospectively analyzed prospectively collected data from neonates born in Muenster University Hospital who underwent early sonographic DDH screening within the first 10 days of life.

Two cohorts were defined based on the screening indication:

- Reference values cohort (2007–2015): Among 1034 neonates (2068 hips) who underwent universal sonographic DDH screening after birth, 25 were screened beyond the 10th day of life, and 78 had incomplete data or underwent imaging in the neonatal intensive care unit (NICU) without adherence to the Graf method. Consequently, 967 neonates (1934 hips) were included. Although this cohort was defined by universal sonographic screening irrespective of risk factors, a proportion of these neonates presented with one or more predefined risk factors (e.g., abnormal birth presentation, positive family history). The presence of risk factors in this cohort, therefore, reflects their natural distribution within the general newborn population rather than a screening indication.
- Risk factor cohort (2015–2022): Among 1989 neonates (3978 hips) who underwent sonographic DDH screening based on predefined risk factors, 36 were screened beyond the 10th day of life, and 53 had incomplete data or underwent imaging in the NICU without adherence to the Graf method. Consequently, 1900 neonates (3800 hips) were included.

**Figure 1:**
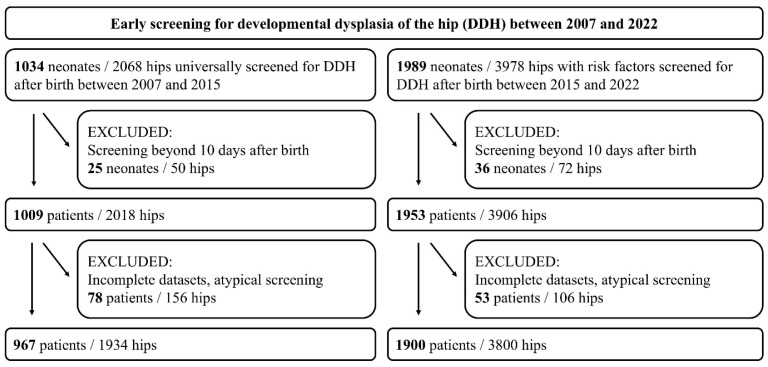
Strengthening the Reporting of Observational Studies in Epidemiology (STROBE) diagram detailing the inclusion and exclusion criteria of the study.

Predefined risk factors included abnormal birth presentation, including breech position, positive family history of DDH, oligohydramnios, multiple gestations, prematurity, congenital foot deformities, and Cesarean section. Abnormal birth presentation was defined as any non-cephalic presentation at delivery, with breech presentation being the most common. A positive family history was defined as the presence of DDH in first-degree relatives (parents or siblings). The study was approved by the ethics committee of the University of Muenster, Germany (ID: 2023-459-f-S). The study findings are reported according to the Strengthening the Reporting of Observational Studies in Epidemiology (STROBE) guidelines (Figure 1) [27].

### 2.2. Sonographic Examination and Diagnostic Criteria

**Figure 2:**
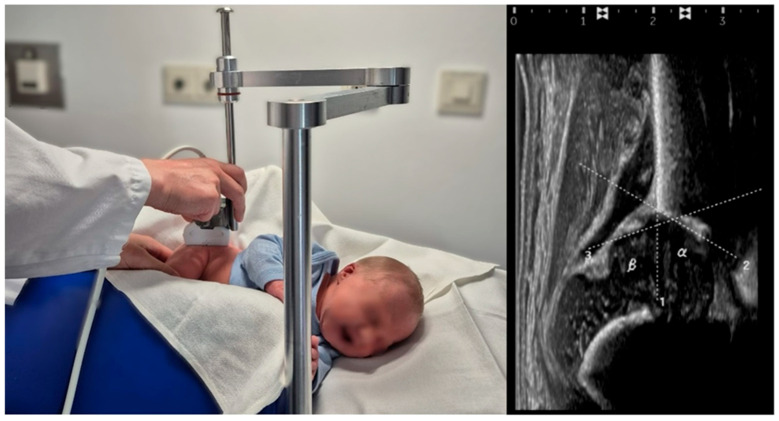
(**Left**): Representative image of the standardized ultrasound examination of a 2-day-old male neonate placed in a left lateral position using a positioning device, according to Graf. (**Right**): Sonographic assessment of the alpha and beta angle of the right hip was performed using the Graf method.

All sonographic examinations were performed using the Graf technique, adhering to standardized protocols. Bilateral alpha and beta angles were measured, and hip maturation status was classified according to Graf’s classification (Figure 2) [7]. DDH was defined as type IIc or worse.

### 2.3. Sample Size and Power Calculation

A power analysis was conducted to ensure an adequate sample size for reference value estimation and risk factor analysis. Assuming a standard deviation (SD) of 5° for the alpha angle and a required precision of ± 1°, at least 96 neonates (192 hips) were needed. For risk factor analysis, an expected mean alpha angle difference of 2° between groups required a minimum of 98 neonates per group (196 total) to achieve 80% power (α = 0.05).

### 2.4. Subgroup Follow-Up Analysis

To evaluate the clinical relevance of borderline alpha angles, we retrospectively identified a subgroup of 20 neonates with alpha angles of 51° or 52° at initial screening. Follow-up sonography was performed after 3–6 weeks.

### 2.5. Statistical Analysis

Baseline characteristics were compared using the Student’s *t*-test for continuous variables and Fisher’s exact test for categorical data. A paired *t*-test was employed to compare parametric paired hip measurements, and a Wilcoxon signed-rank test was used for non-parametric data. The association between risk factors and DDH diagnosis was evaluated using univariate and multivariate logistic regression analysis, with results reported as odds ratios (OR) and 95% confidence intervals (CI), visualized using a forest plot. Variables with a *p*-value < 0.1 in univariate analysis were included in the multivariate model. A *p*-value < 0.05 was considered statistically significant.

Reference ranges were defined using the mean ± 2 SD, corresponding to the central 95% of a normally distributed population. This approach is commonly used in the literature to establish clinically relevant thresholds while accounting for natural biological variability [28,29]. Receiver Operating Characteristic (ROC) analysis was performed to assess the diagnostic performance of the alpha angle, and the Area Under the Curve (AUC) was calculated.

Descriptive statistics, including median and range, were used to summarize the distribution of alpha angles and the changes between initial and follow-up measurements. Clopper–Pearson calculation was applied to estimate the CI for the hip proportion reaching 55° at follow-up.

## 3. Results

Patients in the reference values cohort were examined on average after 2.3 ± 1.3 days, while those in the risk factor cohort were examined after 2.1 ± 1.4 days. The characteristics of the reference values and risk factor cohorts are presented in Table 1.

### 3.1. Sonographic Values

**Figure 3:**
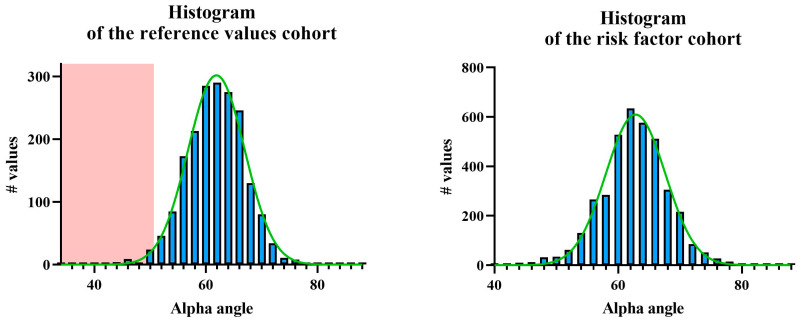
Frequency distribution of alpha angles according to Graf (**left**) in the reference values cohort and (**right**) in the risk factor cohort. The green curve represents the Gaussian normal distribution, while the red area indicates the pathological range, defined as two standard deviations (SD) below the mean.

The mean alpha angle in the reference values cohort was 61.2° ± 5.3°, with a reference range of 50.5–71.9° (Figure 3). The mean beta angle was 70.8° ± 8.6°, with a reference range of 53.6–88.0°. The DDH prevalence was 2.5% (24/967), corresponding to type IIc or higher according to the Graf classification. The alpha angles of the right hips were significantly lower by 1.2° ± 5.3° compared to those of the left hips (*p* < 0.001). The mean alpha angle in the risk factor cohort was 62.1° ± 5.5°. The mean beta angle was 69.2° ± 9.1°. According to Graf, the DDH prevalence was 3.2% (60/1900), with no significant difference compared to the first cohort (*p* = 0.350). The alpha angles of the right hips were significantly lower by 1.3° ± 5.1° compared to those of the left hips (*p* < 0.001). In the ROC analysis of the alpha angles of both cohorts, the AUC was 0.55 with a standard error of 0.008 (*p* < 0.0001). At alpha angle of 50.5°, the sensitivity was 97.5%, while the specificity was 2.4%.

### 3.2. Risk Factor Analysis

**Figure 4:**
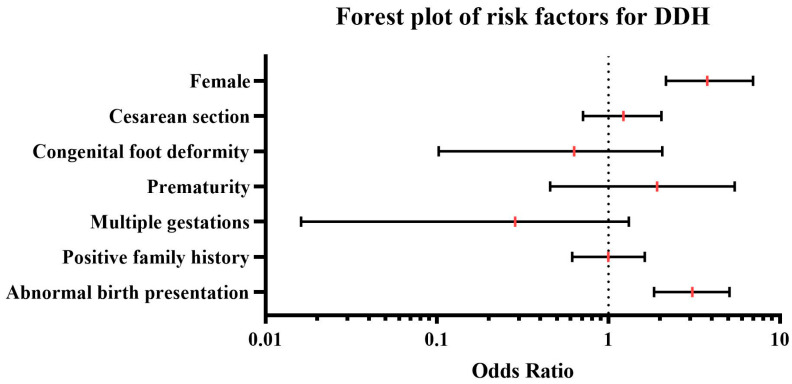
Forest plot illustrating the association between various risk factors and developmental dysplasia of the hip (DDH). The plot displays odds ratios (OR) and corresponding 95% confidence intervals (CI) derived from multivariate logistic regression analysis. The vertical red lines represent the ORs, while the horizontal black lines indicate the 95% CIs. An OR > 1 suggests an increased risk for DDH associated with the respective variable.

Logistic regression analysis revealed a significant association of DDH with abnormal birth presentation (OR = 3.09, 95% CI: 1.84–5.08, *p* < 0.001), while no association was observed with positive family history (OR = 1.00, 95% CI: 0.61–1.63, *p* = 0.995), multiple gestations (OR = 0.29, 95% CI: 0.02–1.31, *p* = 0.126), prematurity (OR = 1.92, 95% CI: 0.46–5.45, *p* = 0.327), oligohydramnios, congenital foot deformity (OR = 0.63, 95% CI: 0.10–2.06, *p* = 0.465), Cesarean section (OR = 1.22, 95% CI: 0.71–2.04, *p* = 0.453). Female neonates were more frequently affected (77.4%) with a significant association with DDH (OR = 3.77, 95% CI: 2.16–6.97, *p* < 0.001). These results were visualized using a forest plot, in which red vertical lines represent the OR and black horizontal lines indicate the corresponding 95% CI (Figure 4).

### 3.3. Follow-Up Subgroup Analysis

Twenty hips with initial alpha angles of 51° or 52° were re-examined after 3–6 weeks. All cases showed significant spontaneous maturation to a minimum of 57° (*p* < 0.001), with a median alpha angle of 61° (range 57–66°) at follow-up. The median increase was 9° (range 6–14°). The available sample achieved a statistical power of 98% to detect a mean increase ≥5°. For the 20 hips, the 95% Clopper–Pearson CI ranged from 83.2% to 100%, corresponding to a high non-maturation rate of up to 16.8%. Based on Clopper–Pearson calculation, at least 72 cases are required to reliably exclude a non-maturation rate >5% with 95% confidence.

## 4. Discussion

Early treatment of DDH is crucial, as prompt intervention can improve hip maturation and potentially prevent long-term complications such as osteoarthritis [4,30]. In Austria, universal early sonographic screening is performed within the first days after birth, emphasizing the importance of identifying abnormal hip development as soon as possible [24,31]. Traditionally, the Graf method was validated for infants during the first 4–6 weeks after birth, and published reference values are based on that age group [7]. The same normal values are also commonly used for early sonography screening, although literature indicates that newborns show considerably different values prior to the postnatal maturation observed later [32]. This may lead to an even higher rate of overdiagnosis and overtreatment in the first days of life, a phenomenon that has already been described in general for sonographic DDH screening [33].

In our study, we provide reference values in neonates for early sonographic screening within the first 10 days of life. The mean alpha angle in the reference cohort was 61.2° ± 5.3°, with a reference range defined as the mean ± 2 SD (50.5–71.9°). This approach of using two SDs below the mean as the pathological cut-off has also been employed due to statistical convention, despite the inherent asymmetry in the distribution of the alpha angle (given the technical upper limit of 90° and the theoretical lower limit of 0°) [28,34]. Toennis et al., in the context of pelvic radiography, described values beyond one SD as requiring follow-up and those beyond two SDs as indicative of more pronounced dysplasia necessitating treatment. Graf et al. cautiously adopted a similar rationale for hip sonography [35,36]. However, a detailed explanation or empirical justification for this practice has not been explicitly provided in either case. Importantly, although the variable is theoretically asymmetric, the observed values in our neonatal cohorts clustered around the mid-range (≈60°), far from the distribution boundaries, and therefore empirically followed a reliable normal distribution. Although the German guideline classifies alpha angles between 50° and 59° in infants under six weeks of age as requiring follow-up but not treatment, our data indicate that, in neonates with a mean age of 2.3 days, these angles fall almost entirely within two standard deviations of the cohort mean [7]. Accordingly, alpha angles in this range should be considered physiological in this age group and no longer indicate the need for further follow-up. In contrast, Li et al. proposed a Z-score-based approach using age- and sex-specific norms, defining pathology by deviation from the mean rather than fixed cut-offs, typically at −2 or −3 SD [37]. However, clinical implementation of this concept is still pending.

Importantly, all current thresholds for alpha angles—whether 50.5° or 60°—are derived from cohort distributions, not from validated diagnostic cut-offs with known predictive power. Even a Graf type I hip (alpha ≥ 60°) does not guarantee long-term morphologic normality, particularly in high-risk populations. Atalar et al. describe in their study 15 patients who had a regular sonography screening, according to Graf, yet were diagnosed with DDH on radiographs at 5 months of age [38].

In our risk factor cohort, the ROC analysis revealed that an alpha angle of 50.5° provided a sensitivity of 97.5% for detecting DDH, although with a specificity of only 2.4%. Given the Gaussian distribution of alpha angles in both the reference and risk factor cohorts, values above 50.5° fall within the upper 97.5% of the observed range and can be considered physiologically normal in the early sonographic screening. These findings support the use of 50.5° as a general cut-off for excluding clinically relevant DDH in neonates, regardless of risk factors. The total number of included patients far exceeds the required sample size based on power analysis, further strengthening the validity of this threshold. Note that the ROC analysis in our study was based on Graf’s established cut-offs for infants aged 4–6 weeks, which we deliberately applied to our neonatal cohort. This approach demonstrates the expected high sensitivity, indicating that all hips classified as normal by Graf are also included in our data, and even more hips fall within the physiological range. The low specificity, in turn, reflects the methodological limitation of using Graf’s thresholds in a younger age group rather than a true diagnostic shortcoming. Accordingly, the ROC results are presented only for comparison with Graf’s values, while the actual neonatal threshold of 50.5° is derived from the variance of our measurements (mean ± 2 SD) and slightly supported by the follow-up subgroup. This underlines our intention to define age-specific reference values that help prevent overdiagnosis and unnecessary follow-up in the neonatal period. Moreover, the ROC analysis yielded an AUC of 0.55, indicating that the alpha angle alone has only limited discriminatory ability in the first 10 days of life. Although this value reached statistical significance due to the large sample size, it does not translate into clinically relevant discriminatory power. This is explained by the application of Graf’s 4–6-week criteria to a neonatal cohort and by the physiological immaturity of the hip in the immediate postnatal period, which results in substantial overlap of values. The ROC results are therefore presented for comparison with Graf’s criteria, while the neonatal threshold of 50.5° is based on the distribution of our measurements and supported by the follow-up subgroup.

Interestingly, we observed significantly lower alpha angles on the right side compared to the left in both cohorts. In contrast, Liu et al. reported slightly higher alpha angles on the right side (+0.8°) in healthy infants [32], and Toennis described higher acetabular index values on the right compared to the left side based on radiographic measurements [35]. The reasons for these divergent findings remain unclear. Possible explanations include true biological variation with side-specific maturation, methodological aspects such as inter- and intraobserver variability of at least 2° inherent to Graf’s method [39], and the fact that our analysis was based on group means rather than paired comparisons. Small but statistically significant differences may therefore lie within the margin of measurement error when analyzing a high number of hips. Taken together, the available evidence indicates side-specific differences but leaves uncertainty regarding their biological significance.

Furthermore, logistic regression analysis confirmed a significant association between DDH and abnormal birth presentation (OR = 3.09) as well as female sex (OR = 3.77), which is consistent with previous literature [16,40,41]. Other risk factors, such as Cesarean section, familial predisposition, and multiple gestations, did not show a significant association with DDH in our analysis.

Notably, DDH prevalence did not differ significantly between the risk-based and universal cohorts. This finding challenges the selectivity of current early screening algorithms and supports the consideration of universal early sonographic screening, as implemented in Austria. Late detection and subsequent surgical intervention rates have been shown to decline with broader screening coverage [2,12].

Additionally, we analyzed a subgroup of 20 hips with borderline alpha angles of 51–52° at initial screening and performed a follow-up ultrasound after 3–6 weeks. All hips showed spontaneous maturation without treatment, with a minimum alpha angle of 57° and a median of 61° at follow-up, corresponding to a median increase of 9° (range 6–14°). The available sample achieved a statistical power of 98% to detect a mean increase ≥ 5°, supporting the robustness of the observed maturation. However, the 95% Clopper–Pearson CI ranged up to 16.8% possible non-maturation, underlining the limited certainty of conclusions based on this small cohort. To exclude a non-maturation rate > 5% with 95% confidence, at least 72 cases would be required. It should also be noted that ultrasound precision for the alpha angle according to Graf is approximately ± 1° on correctly acquired standard planes [36], while inter- and intraobserver variability may reach ~2–4°, with reported SDs of about 5° across studies [41]. Small side differences or marginal increases may therefore partly fall within the range of measurement error. Taken together, these findings indicate a trend that alpha angles ≥50.5° in the neonatal period represent physiological immaturity rather than structural pathology, consistent with the observations reported by Tschauner et al. [4]. Nonetheless, larger prospective studies are necessary to confirm this observation.

Several limitations should be noted. First, the risk factors were primarily determined by information provided by obstetricians and through parental interviews at admission; hence, incomplete documentation cannot be ruled out. This reliance on external documentation and self-reported information may also have introduced misclassification bias, which, together with the retrospective study design, limits the strength of causal inferences. Second, the sonographic measurements were performed by different experienced examiners without a formal interobserver reliability analysis. A recent meta-analysis by Quader et al. [41] reported up to 25% variability in alpha angle measurements, emphasizing the potential for systematic differences between observers and thereby limiting the reproducibility of our findings. Third, the study period extended from 2007 to 2022, during which technical advances in ultrasound equipment, examiner experience, and referral practices may have occurred. These potential era effects could have influenced the measurements and may limit direct comparability across cohorts. Fourth, the lack of systematic follow-up data for patients referred to local pediatricians after initial screening is a limitation, resulting in a follow-up cohort of only 20 patients, without the possibility of drawing a reliable conclusion. Finally, the retrospective analysis of prospectively collected data precludes the ability to assess the longitudinal predictive value of early sonographic findings regarding the eventual development of DDH.

## 5. Conclusions

Early sonographic screening for DDH within the first 10 days of life enables the definition of age-specific reference values and supports a 50.5° alpha angle cut-off to differentiate physiological maturation from pathological dysplasia. Abnormal birth presentation and female sex emerged as robust risk factors, whereas the lack of association with Cesarean section and familial predisposition challenges their diagnostic relevance in the neonatal period. Notably, in our small subcohort of 20 patients, all hips with initial alpha angles of 51–52° showed reliable spontaneous maturation at follow-up. These findings provide a foundation for risk-adapted screening strategies and emphasize the need for follow-up studies to evaluate the longitudinal predictive value of early sonographic findings.

## Figures and Tables

**Table 1:** Characteristics of patients of the reference values and risk factor cohorts (f = female; SD = standard deviation; abnormal birth presentation = any non-cephalic presentation). In the reference values cohort, risk factors are listed to reflect their prevalence in the general newborn population; they were not the indication for screening in this group.

Characteristics	Reference Values Cohort	Risk Factor Cohort
Patients no. (f %)	967 (47.7%)	1900 (49.4%)
Age (days ± SD)	2.3 ± 1.3	2.1 ± 1.4
Risk factors (%)	352 (36.4%)	1900 (100%)
- Abnormal birth presentation - Positive family history - Oligohydramnios - Multiple gestations - Prematurity - Congenital foot deformity - Cesarean section	54 (5.6%) 291 (30.0%) 0 (0%) 19 (2.0%) 0 (0%) 27 (2.8%) 291 (30.0%)	321 (16.9%) 1006 (52.9%) 3 (0.2%) 92 (4.8%) 46 (2.4%) 91 (4.7%) 511 (26.9%)
Developmental dysplasia of the hip (%)	24 (2.5%)	60 (3.2%)

## Data Availability

The data presented in this study are available on request from the corresponding author.
